# Computational Modeling of Seizure Dynamics Using Coupled Neuronal Networks: Factors Shaping Epileptiform Activity

**DOI:** 10.1371/journal.pcbi.1004209

**Published:** 2015-05-13

**Authors:** Sebastien Naze, Christophe Bernard, Viktor Jirsa

**Affiliations:** 1 UMR1106 Inserm, Institut de Neurosciences des Systèmes, Marseille, France; 2 Aix-Marseille University, Marseille, France; UFR Biomédicale de l’Université René Descart, France

## Abstract

Epileptic seizure dynamics span multiple scales in space and time. Understanding seizure mechanisms requires identifying the relations between seizure components within and across these scales, together with the analysis of their dynamical repertoire. Mathematical models have been developed to reproduce seizure dynamics across scales ranging from the single neuron to the neural population. In this study, we develop a network model of spiking neurons and systematically investigate the conditions, under which the network displays the emergent dynamic behaviors known from the Epileptor, which is a well-investigated abstract model of epileptic neural activity. This approach allows us to study the biophysical parameters and variables leading to epileptiform discharges at cellular and network levels. Our network model is composed of two neuronal populations, characterized by fast excitatory bursting neurons and regular spiking inhibitory neurons, embedded in a common extracellular environment represented by a slow variable. By systematically analyzing the parameter landscape offered by the simulation framework, we reproduce typical sequences of neural activity observed during status epilepticus. We find that exogenous fluctuations from extracellular environment and electro-tonic couplings play a major role in the progression of the seizure, which supports previous studies and further validates our model. We also investigate the influence of chemical synaptic coupling in the generation of spontaneous seizure-like events. Our results argue towards a temporal shift of typical spike waves with fast discharges as synaptic strengths are varied. We demonstrate that spike waves, including interictal spikes, are generated primarily by inhibitory neurons, whereas fast discharges during the wave part are due to excitatory neurons. Simulated traces are compared with in vivo experimental data from rodents at different stages of the disorder. We draw the conclusion that slow variations of global excitability, due to exogenous fluctuations from extracellular environment, and gap junction communication push the system into paroxysmal regimes. We discuss potential mechanisms underlying such machinery and the relevance of our approach, supporting previous detailed modeling studies and reflecting on the limitations of our methodology.

## Author Summary

Neurons communicate via different types of synapses on very fast time scales. The combination of hundred thousand of such interconnected cells within a fluctuating extracellular environment forms a complex network that gives rise to function and behavior via the formation of dynamical patterns of activity. In the context of epilepsy, the functional properties of the network at the source of a seizure are disrupted by a possibly large set of factors at the cellular and molecular levels. It is therefore needed to sacrifice some biological accuracy to model seizure dynamics in favor of macroscopic realizations. Here, we present a neuronal network model that convenes both neuronal and network representations with the goal to describe brain dynamics involved in the development of epilepsy. We compare our modeling results with animal *in vivo* recordings to validate our approach in the context of seizures. Such system-level methodology has significant bearing in understanding neuronal network dynamics that entangle multiple synaptic and extracellular modalities.

## Introduction

Epilepsy is characterized by seizures, a paroxysmal behavior that results from abnormal, excessive or hypersynchronous neuronal activity in the brain [1], with a various set of symptomatic outcomes depending on brain regions involved in its generation and propagation processes. Clinically, epilepsy affects 1% of the population, from whom 30% are drug-resistant. Physiological investigations of neural tissue in the context of human Temporal Lobe Epilepsy and experimental models revealed neuronal loss in the hippocampus, rewiring of excitatory and inhibitory pathways [2], in keeping with the hypothesis on unbalanced excitation/inhibition ratio observed in epilepsy [3,4].

Understanding seizure mechanisms from micro to macro scales is necessary to provide clinicians and basic scientists with a reliable theoretical basis to develop new therapeutic approaches. Computational modeling reproducing brain activity is a genuine approach to investigate such multi-scale paradigms. Neural network models in the context of epilepsy typically use multi-compartment Hodgkin-Huxley type neurons with a collection of ion-channels dynamics and multiple excitatory and inhibitory synaptic combinations. We will here refer to them as biophysically-realistic, see for example [5,6]. Reduced population models (so-called neural masses or mean field models) absorb a significant amount of biophysical details in constant parameter values and are referred to as large-scale or macroscopic [4,7–9], see for a review [10]. A third type of modeling scheme consists in approaching the dynamics of seizures in an abstract manner, and describing them in terms of generic dynamic features [11]. The advantage of this approach is its generality, allowing the identification of invariant seizure classes based on basic dynamical properties. The drawback lies in the difficulty to find biophysical correlates to the state variables used in such an approach. Certain elementary features such as dynamics evolving on different time scales guides the identification of the biophysical correlates. For example, recent emphasis in seizure modeling is directed towards the role of extracellular environmental fluctuations, which evolve on a significantly slower time scale than neuronal discharges. By incorporating slow extracellular potassium or oxygen levels as key parameters, state-of-art studies displayed transitions between pathological brain states observed during paroxysmal activity [12–14]. Such approaches combine dynamical systems theory and large-scale neural network computations to propose key insights into seizure mechanisms. However, extracellular potassium homeostasis provides only a partial answer. Many different biophysical factors can lead to seizure genesis [15,16], in keeping with the concept that different parameter sets can produce the same type of activity at the network level [17]. Introducing all those parameters in a detailed model poses a computational and theoretical challenge, but may be only useful if the arising network behavior can be characterized dynamically.

**Fig 1:**
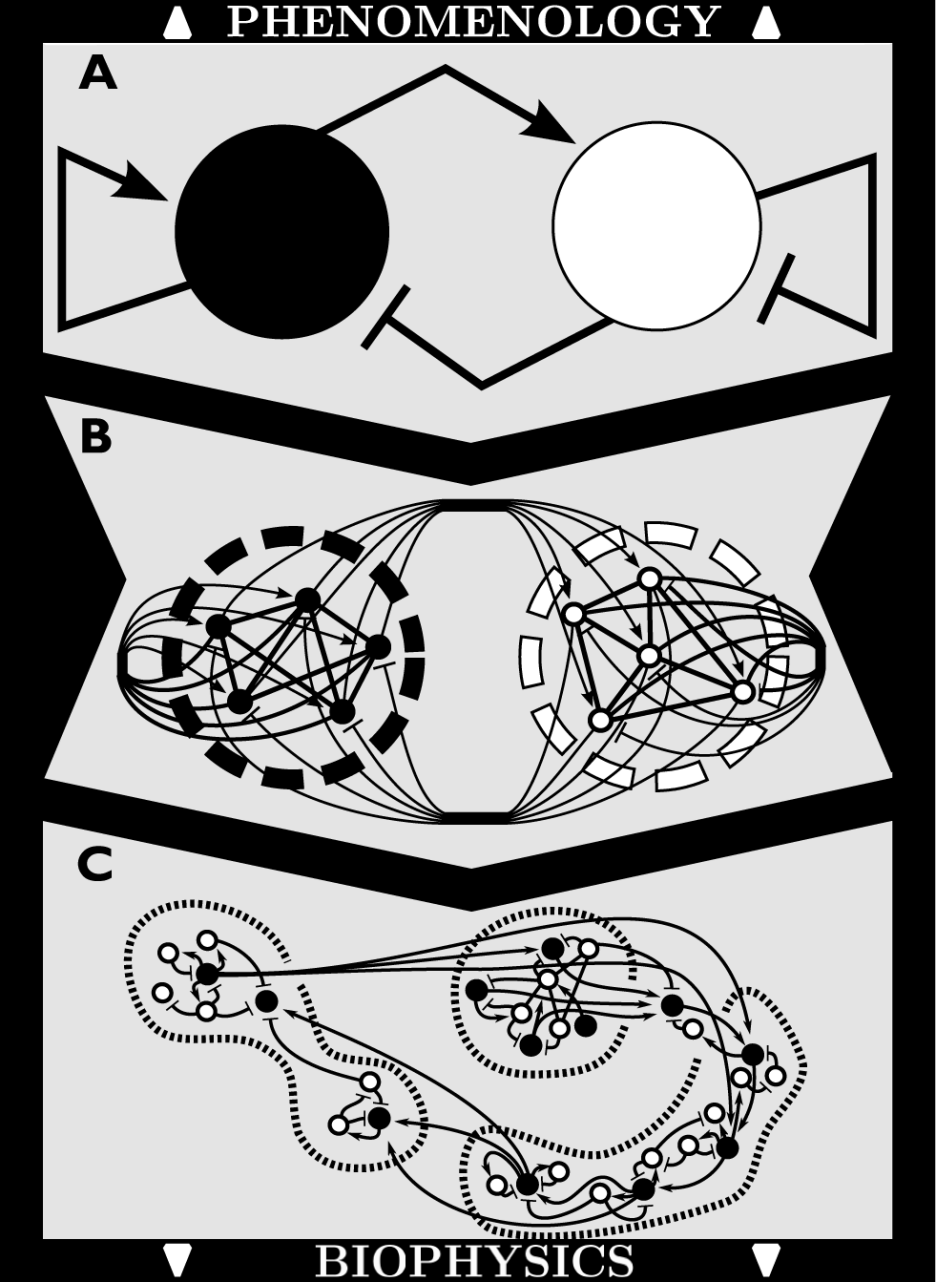
Multiscale top-down approach in modeling neural networks, from phenomenological to biophysical architectures. A) Neural mass model composed of excitatory (black circle) and inhibitory (white circle) populations, affected by surrounding environment (grey background). Populations act on each other via linear transfer functions, with positive gain for excitatory couplings (arrow heads) and negative gain for inhibitory couplings (flat heads). B) Single neurons (dots) are enclosed within each population (thick dashed circles). Black dots are excitatory neurons and white dots are inhibitory neurons. Linear gap junction coupling is included between neurons from the same genre (i.e. straight lines on exc—exc and inh-inh connections). Non-linear synaptic coupling is introduced within and between populations (curved lines) in a fully-connected topology. C) Excitatory and inhibitory single neurons (black and white dots) are distributed spatially in a biologically relevant topological structure (thin dashed ensembles), each of which being a subset from B).

In the present study, we make the link of the seizure state dynamics across neuron and population levels explicit. **Fig 1** illustrates the line of thought deriving the here presented intermediate system architecture (panel B) from the phenomenological model (panel A); future work could comprise spatially structured networks of neurons (panel C). We specifically develop a network of inhibitory spiking and excitatory bursting neurons, driven by a slow environmental variable, reproducing characteristic features of the temporal evolution of human and experimental seizures. Inspired by the phenomenological model of spontaneous seizure generation from [11], the so-called Epileptor, and mean field dimension-reduction techniques [18], we derive the former equations as introduced in [11] from more biophysically-inspired representations of the system, including single neuron dynamics, linear and non-linear synaptic interactions portraying gap junctions and chemical synapses, respectively. Meanwhile adding this new level of complexity, we keep track of the emergent network behavior exhibited in the abstract model by systematically exploring dynamical changes induced by parameter sweeps in our new system. Our slow environmental variable is more abstract than the biophysically explicit extracellular milieu and oxygenation as considered in [12–14] and involves a wider set of physiological factors such as intra/extracellular pH ratio, oxygen availability, extracellular potassium, calcium, and chloride concentrations, for example. These parameters have been studied experimentally and demonstrated to be part of the influencing agents leading to seizures [3]. Introducing these variables into existing detailed biophysical models has been subject to great consideration for the last decade [19–22]. The abstract integration of such local mechanisms into a slow environmental variable, as presented here, aims at describing the slow sub-system from a more conceptual perspective.

Following this direction, large scale simulations of neural systems become possible at reasonable computational cost, but with sufficient accuracy to treat complex dynamical mechanisms, such as multi-clustered synchronization or entrainment, while implying biologically realistic synapses and firing patterns. We systematically identify synchronization regimes and reproduce potential routes through status epilepticus (by definition, a seizure lasting more than 20 minutes) in our parameter space, providing interpretation of its underlying neuronal mechanisms and further validating our model. We accredit the different parametric regimes experimentally against rodent data recorded *in vivo* and demonstrate that the different routes in parameter space are consistent with the theoretically predicted topology. Then, exploring a regime that generates spontaneous seizures from background activity, we infer new insights from the role of excitatory and inhibitory synapses, as well as the extracellular environment.

## Materials and Methods

### Experimental procedures

#### Ethics statement

All experimental protocols were approved by the French Institute of Health and Medical Research (INSERM).

#### Epidural recordings

Recordings were performed 24/7 on adult male Wistar rats (n = 5). A recording skull screw was secured above hippocampal CA1 (-4.0 mm posterior, +2.0 mm lateral), and a reference skull screw was secured above cerebellum. The leads of the telemetry probe (Data Science International (DSI), St. Paul, MN, USA) were wrapped around the recording and reference screws and screws were all encased in dental cement. Rats were allowed 7 days post-surgical recovery before any further experimental procedures were conducted. Status epilepticus (SE) was induced with kainic acid injected once per hour at a dose of 5 mg/kg until SE was observed. After 30 minutes of SE, rats received an ip injection of diazepam (8 mg/kg).

#### Data analysis

SE and spontaneous seizures were detected and analyzed using Clampfit (Molecular Device) software.

### Epileptor

The Epileptor [11] is a five-dimensional model and comprises three different time scales accounting for various electrographic patterns: On the fastest time scale, two state variables (ensemble 1) exhibit bistable dynamics between oscillatory activity modeling fast discharges and a stable node representing interictal activity. On the intermediate time scale, two state variables (ensemble 2) model the spike and wave events (SWE) and form the second neuronal ensemble. On the slowest time scale (order of tens of seconds), the evolution of a very slow permittivity variable guides the neural population through the seizures including seizure onset and offset. The first ensemble is linearly inhibited by the second ensemble in order for fast discharges to occur only during the wave part of the SWE, the second ensemble is excited by the first ensemble through a low-pass filter coupling in order to generate SWE and interictal spikes. Both ensembles are coupled through the permittivity variable. A separatrix divides the state space of the first ensemble between ictal and interictal states and acts as a barrier. As the permittivity variable evolves over time, seizure onset occurs through a saddle-node bifurcation showing a direct current (DC) shift at the transition between the interictal and the ictal state. Seizure offset occurs through a homoclinic bifurcation showing the logarithmic scaling of interspike intervals when approaching seizure offset. The time course of the local field potential is related to the total activity of both ensembles. A more detailed description of the model can be found in Jirsa et al [11], with an extended analysis of its embedded dynamics in [23].

### Neuronal models

We model the dynamics of brain activity across a set of two populations P_1_ (excitatory) and P_2_ (inhibitory) of *N* neurons each, x_1,j_ and x_2,j_, respectively (j∈[1,N]). The dynamics of the population P_i_ is determined by ${\bar{x}}_{i}=\frac{1}{N}\sum_{j=1}^{N}x_{i,j}$ as described by the mean field approximation used for neural masses [24], and the dynamics of membrane potentials x_i_ (i∈[1,2]) is defined by Hindmarsh-Rose (Eq (1.1), (1.2) and (1.3)) and Morris-Lecar (Eq (2.1), (2.2), (2.3), (2.4) and (2.5)) neuronal models for P_1_ and P_2_, respectively, as follows. For sake of clarity, the j^th^ index is omitted in the notation. Our choice of the Hindmarsh-Rose model is motivated by the fact that its phase flow is isomorphic to the phase flow of the first Epileptor ensemble [11]. This neuron model is a square-wave burster governed by saddle-node and homoclinic bifurcations, and so is the first Epileptor ensemble [25]. Similarly, the Morris-Lecar model, via its saddle-node-on-invariant-circle (SNIC) bifurcation, captures the same excitable properties as the second Epileptor ensemble.

*Population 1 neuron (Hindmarsh-Rose)*:

$${\dot{x}}_{1}=y_{1}-ax_{1}^{3}+bx_{1}^{2}-z+I_{1}+C_{E}({\bar{x}}_{1}-x_{1})+\sigma_{1}I_{syn}({\bar{x}}_{1},x_{1})+\sigma_{1}I_{syn}({\bar{x}}_{2},x_{1})+W(t)$$ (1.1)

$${\dot{y}}_{1}=c-dx_{1}^{2}-y_{1}$$ (1.2)

$$\dot{z}=r(s_{1}(x_{1}+{\bar{x}}_{2}-x_{0})-\bar{z})$$ (1.3)

*Population 2 neuron (Morris-Lecar)*:

$$C_{M}\dot{V}\;=\;I_{2}-g_{L}\left(V-E_{L}\right)-g_{K}n\left(V-E_{K}\right)-g_{Ca}m_{\infty }\left(V\right)\left(V-E_{Ca}\right)+\sigma_{2}C_{E}({\bar{\text{x}}}_{2}-{\text{x}}_{2})+I_{syn}({\bar{x}}_{1},x_{2})+I_{syn}({\bar{x}}_{2},x_{2})-\sigma_{2}\times 0.3(\bar{z}-3)+\sigma_{2}W\left(t\right)$$ (2.1)

$$\dot{n}\;=\;\phi \left(n_{\infty }\left(V\right)-n\right)/\tau_{n}\left(V\right)$$ (2.2)

*with*

$$m_{\infty }\left(V\right)\;=\;\frac{1}{2}\left[1+\text{tanh}\left(\left(V-V_{1}\right)/V_{2}\right)\right]$$ (2.3)

$$\tau_{n}\left(V\right)\;=\;1/\text{cosh}\left(\left(V-V_{3}\right)/2{\text{V}}_{4}\right)$$ (2.4)

$$n_{\infty }\left(V\right)\;=\;\frac{1}{2}\left[1+\text{tanh}\left(\left(V-V_{3}\right)/V_{4}\right)\right]$$ (2.5)

*and*

$$x_{2}=\frac{V}{20}$$

Coupling term C_E_ and function I_syn_ are detailed in a next section. σ_i_ is a scaling ratio between the two pools of neurons in order to have similar membrane potential amplitudes across different neuron types (i∈[1,2]). Parameters are the same as in previously published studies [5,24,25], unless otherwise mentioned. They are in part enumerated in **Table 1** together with their biophysical interpretation, when applicable. W(t) is white Gaussian noise of uniformly distributed values in the interval [-W_max_,W_max_] where W_max_ ranges from 2.5 to 20 mV as provided in Table 1, 2.5 mV being used for lowest noise simulations and 20 mV for highest noise simulated traces. Here, all simulations were performed with bounding value of W_max_ at 6 mV unless stated otherwise. The other parameters are constant and their ranges are included in Table 1. All values are set at the initialization of the simulation and remain constant for the time of the run.

**Table 1:** List of parameters (ranges spanned for parameter space exploration of Fig 3 are indicated under squared brackets, N.A. stands for “Not Applicable”).

*Parameter*	*Description*	*Value*
***Population 1 parameters***		
a	N.A.	1.0
b	N.A.	3.0
c	N.A.	1.0
d	N.A.	5.0
s_1_	N.A.	8.0
I_1_	Baseline input current	3.1
***Global parameters***		
N	Number of neurons per population	40
x_0_	Degree of epileptogenicity	[-4.5, -2.5]
r	slow timescale constant	[0.000004, 0.00002]
W_max_	Maximum absolute amplitude of the white noise process W(t)	[1, 20]
***Coupling parameters***		
C_E_	Normalized electrical coupling strength	[0, 1]
G_s_^i,i^	intra-population synaptic coupling (μS)	[0, 0.4]
G_s_^i,j^	inter-population synaptic coupling (i≠j) (μS)	[0, 0.4]
*σ*_1_	Scaling factor for upscaled inputs towards P_1_	1/50
*σ*_2_	Scaling factor for downscaled inputs towards P_2_	50
T_max_	Maximum concentration of transmitter in the synaptic cleft (mM)	1
α_e_	Forward binding rate constants of the excitatory synapses to open the receptors (mM^-1^ msec^-1^)	1.1
α_i_	Forward binding rate constants of the inhibitory synapses variable T to open the receptors (mM^-1^ msec^-1^)	5
β_e_	Backward binding rate constants of the excitatory synapses to close the receptors (msec^-1^)	0.19
β_i_	Backward binding rate constants of the inhibitory synapses to close the receptors (msec^-1^)	0.18
V_t_	Value at which the transmitter release function is half-activated (mV)	2
K_p_	Steepness of the transmitter release function exponential (mV)	5
E_e_	Excitatory synapses reversal potential (mV)	0
E_i_	Inhibitory synapses reversal potential (mV)	-80
***Population 2 parameters***		
C_M_	Membrane capacitance (μF/cm^2^)	20.0
I_2_	Baseline input current (μA/cm^2^)	40.0
V_1_	Potential at which *m*_*∞*_ = 0.5 (mV)	1.2
V_2_	Reciprocal of slope of voltage dependence of *m*_*∞*_ (mV)	18.0
V_3_	Potential at which *n*_*∞*_ = 0.5 (mV)	12.0
V_4_	Reciprocal of slope of voltage dependence of *n*_*∞*_ (mV)	17.4
*ϕ*	Maximum rate constant for calcium channel opening (s^-1^)	0.067
E_Ca_	Calcium ion channels reversal potential (mV)	120
E_K_	Potassium ion channels reversal potential (mV)	-84
E_L_	Leak current reference potential (mV)	-60

I_1_, I_2_ are baseline input currents of neurons for populations 1 and 2, respectively. x_0_ captures the equilibrium point of the permittivity z (Eq 1.3) and has previously been referred to as degree of epileptogenicity [26] in the context of the Epileptor. As mentioned above, it corresponds to the mutual effect of a set of factors influencing neural excitability including ATP availability, oxygenation, extracellular potassium concentration etc.

### Coupling through electrical and chemical synapses

We use symmetric linear difference coupling across membrane voltage equations on fast time scales to model gap junctions, also referred to as electrical synapses. This type of communication, being only electrically conductive through the cell's membrane, is considered nearly instantaneous so its transmission delay is negligible. It is described in the equations by the difference $C_{E}\left({\bar{x}}_{i}-x_{i}\right)$ where ${\bar{x}}_{i}$ refers to the mean activity of the neural population *i*, and *C*_*E*_ to the coupling strength. Note that when all neurons from a population are synchronized this difference is zero. Only neurons belonging to the same population are connected to each other via such linear coupling as it is observed in neural tissue that gap junctions usually connect neurons from the same class [27].

Chemically mediated synaptic transmissions follow more complex dynamics induced by intermediate biological mechanisms such as pre-synaptic neurotransmitter release, post-synaptic receptor binding, G-protein activation and so on. Models of such dynamics are typically non-linear and invoke physiologically most relevant parameters, including transmitters’ concentration and release time, conductance strength, or re-uptake time. We used the model described in [5,28], as it provides a sufficiently accurate level of biological description for our study.

The equations are:

$$I_{syn}(x_{i},x_{j})=-G_{S}^{i,j}u(x_{j}-E\;)$$ (3.1)

$$\dot{u}=\alpha T(1-u)-\beta u$$ (3.2)

$$T=\frac{T_{\text{max}}}{1+e^{-(x_{i}-V_{t})/K_{p}}}$$ (3.3)

where *I*_*syn*_ is the post-synaptic current, *x*_*i*_ and *x*_*j*_ are the pre- and post-synaptic neuron activity, respectively. u is an auxiliary variable for the computation of the post-synaptic current, E is the reversal potential, G_s_^i,j^ the conductance strength of synapses from neurons of population i to neurons from population j, with (i,j) ∈ [1,2]. α, β the forward and backward binding rate constants with transmitter concentration *T* in the synaptic cleft, of which the maximum is set by the constant *T*_*max*_. *K*_*p*_ gives the steepness and *V*_*t*_ sets the value at which the function is half-activated. We note that dendritic spatial summation corresponds to the process, in which the input *x*_*i*_ is averaged over the whole population when sent to the synapse’ function I_syn_.

### Metrics

EEG time series of epileptic seizures display a large diversity of temporal characteristics such as interictal spikes, ripples, tonic/clonic discharges etc. It is therefore non-trivial to define a precise measure that delineates these features with sufficient accuracy. To analyze the output of our neuronal populations, we consider an index of synchronization between oscillators: the Kuramoto Order Parameter (KOP) [29]. To visualize the dynamics of the phases of each neuronal oscillator, it is convenient to imagine a swarm of points tracing out the unit circle in the complex plane when action potentials occur. The complex order parameter [30] is the norm of the sum of all vectors between the origin of the unit circle and the points around the circle. It is defined as follow:

$$re^{i\psi }=\frac{1}{N}\sum_{j=1}^{N}e^{i\theta_{j}}$$ (4.1)

The norm *r* of this macroscopic quantity is near zero when action potentials are uniformly distributed over time, and increases as firings get synchronized. An animated version of these motions is presented in **S1 Movie**. It can be interpreted as the collective rhythm produced by the whole population. Although it is a convenient method to quantify synchrony, this measure is applicable only when oscillations are present. Neurons at rest (i.e. not oscillating) have been considered separately and labeled explicitly at rest in the analysis. Special care must be taken while interpreting our synchronization measure: we do not consider the synchronization between P_1_ and P_2_ but rather the synchronization within P_1_ (KOP_1_) and within P_2_ (KOP_2_). The overall KOP is calculated as the sum of KOP_1_ and KOP_2_.

### Simulations

The code is written in Python with an object-oriented architecture, of which an online version is made available together with its documentation at the Github repository (https://sebnaze@bitbucket.org/sebnaze/epilepton.git). Simulations were performed on a parallel computing cluster using the Euler-Maruyama integration scheme [31] with step size dt = 0.05.

## Results

In this section, we first present how a network of spiking and bursting neurons is derived from the phenomenological Epileptor model, using methods from dynamical systems theory. Then, numerical simulations of the model jointly with experimental data in the context of seizures are exposed. 40 excitatory and 40 inhibitory neurons arranged in a fully synaptically connected network with clustered gap junction communications were used to simulate relevant biophysical mechanisms. With this setup, we explored a set of physiologically pertinent parameters. After comparing simulated data with experimental recordings from animal models of epilepsy, we propose sequential stages that may underlie status epilepticus and validate our model. Further investigating the role of synaptic coupling in a regime generating spontaneous seizures, we make predictions of seizure profiles in the context when synaptic communication is not present.

### A. Modeling: From Epileptor to a network of spiking and bursting neurons

**Fig 2:**
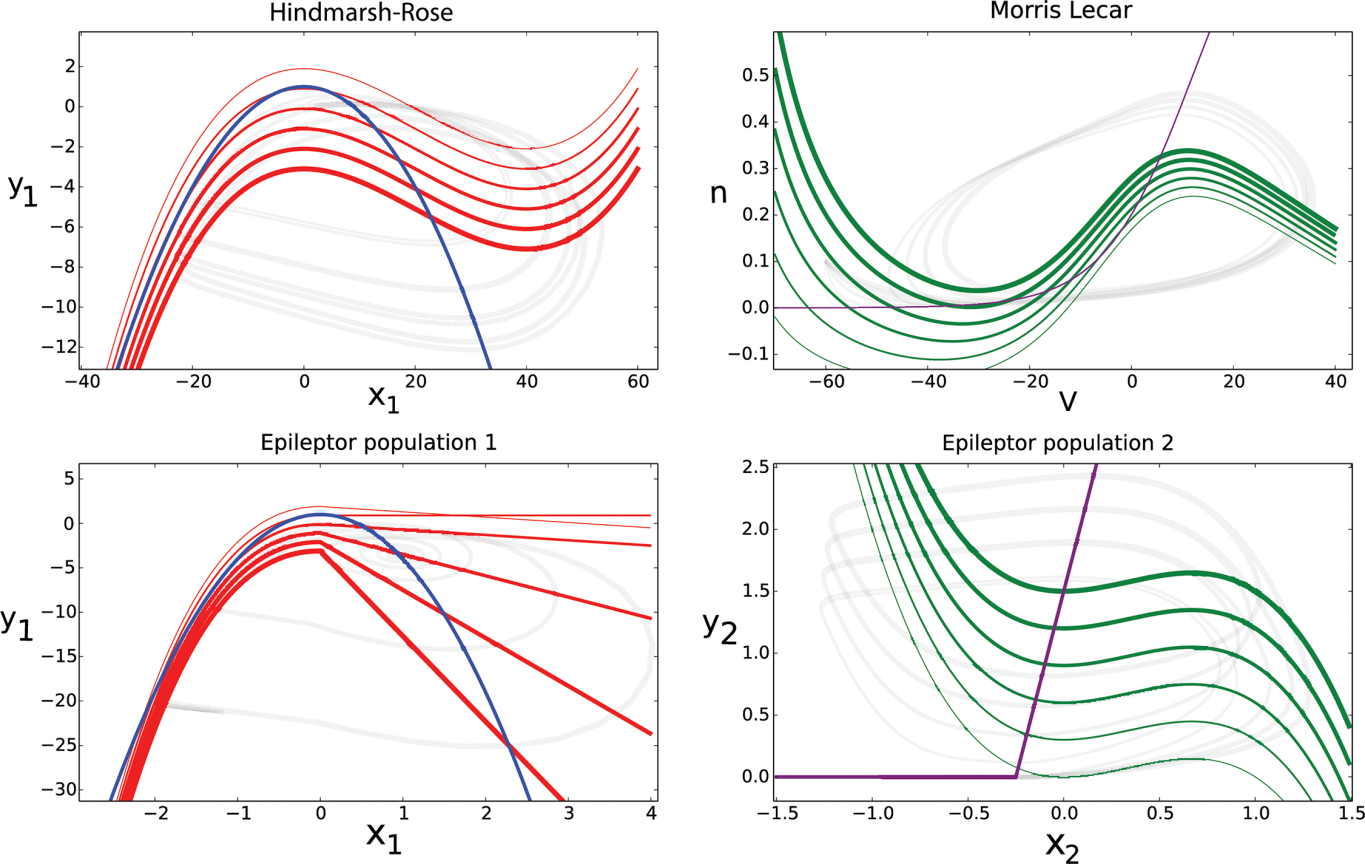
Comparison of phase space topologies between single neuron models and the Epileptor ensembles. Top row shows the null clines of the Hindmarsh-Rose model (Eq 1.1 and 1.2) in two-dimensions (left) and the Morris-Lecar model (right, Eq 2.1 and 2.2). Bottom row shows the null clines of Epileptor ensemble 1 (left) and Epileptor ensemble 2 (right). Values of the z variable range from 0 to 5, thicker lines correspond to lower z. As nullclines intersect, fixed points are created. Topological equivalence of phase flows is preserved in all conditions.

To build a system dynamically isomorph to the Epileptor model [11], but with a link to biophysical properties, we constructed a network of Hindmarsh-Rose bursting and Morris-Lecar spiking model neurons. **Fig 2** illustrates the dynamical similarities between the neuron models and the original Epileptor ensembles, and provides a comparison of their phase space topologies for various levels of excitation. In the Epileptor, the first ensemble shows a saddle-node bifurcation at seizure onset and a homoclinic bifurcation at seizure offset. The Hindmarsh-Rose model is a square-wave burster also governed by saddle-node and homoclinic bifurcations [25] and thus isomorph regarding its phase flow with the first Epileptor ensemble [11] (**Fig 2**, left column). The spike-wave discharge is modeled in the second Epileptor ensemble by a saddle-node on invariant cycle (SNIC) bifurcation, which is also present in the Morris-Lecar model [25] (**Fig 2**, right column). When the excitatory and inhibitory spiking neurons are electrically coupled within the populations via gap junctions, then synchronization occurs. By construction, the synchronization manifold is identical to the uncoupled neuron models and thus has the same bifurcations and phase flow topology as the Epileptor ensembles. This is valid precisely for full synchronization and approximately for partial synchronization as a function of the coupling strength. Due to the dynamic isomorphism of single neuron models and the Epileptor ensembles, when full synchronization is approached the mean field of the populations expresses the full dynamic range of behaviors known from the Epileptor model. This will be shown in the next section, in which a parameter space analysis maps out the synchronization behaviors to large detail.

### B. Status epilepticus: The route towards spontaneous seizures

**Fig 3:**
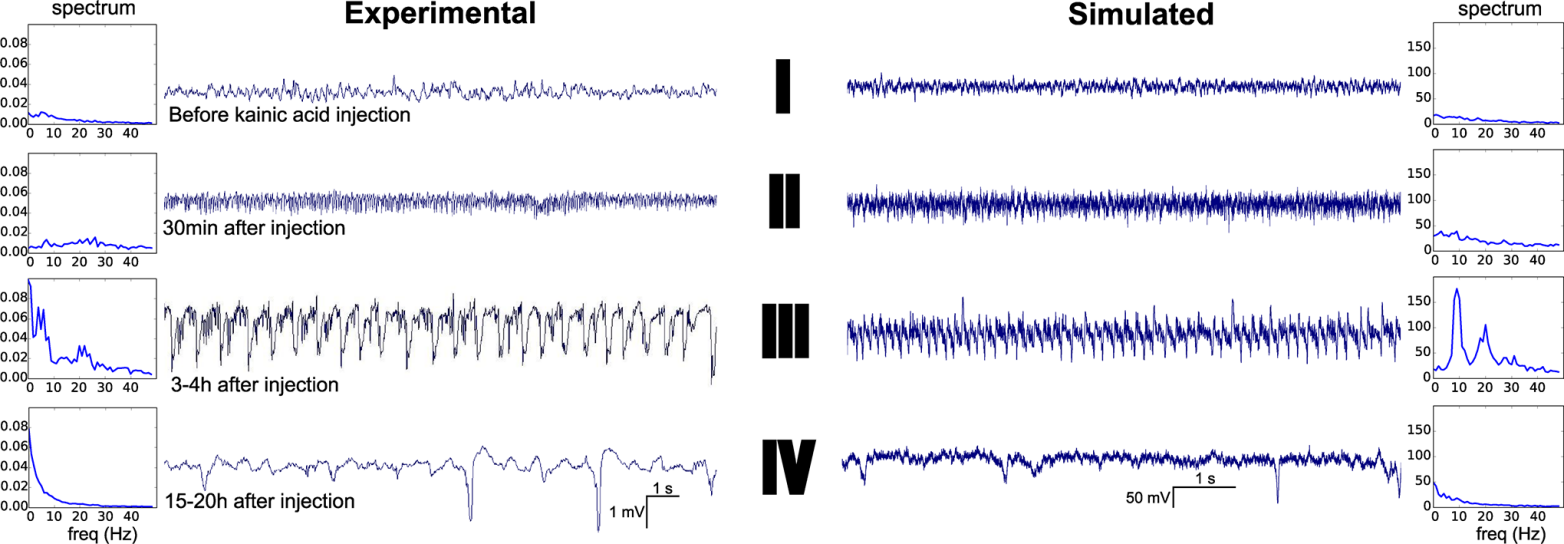
The different phases of status epilepticus, experimental (left) and simulated (right) traces, with corresponding spectra. Simulated traces are plotted as the weighted sum of the populations’ activity, being 80% excitatory and 20% inhibitory. One population activity is the mean of its neurons membrane potentials. Simulated and experimental signals are band-pass filtered at 1–50 Hz (x-axis of the spectra). Power spectral densities (in arbitrary units) are computed using a fast Fourier transform of signals over 20s duration and sampled at 256 Hz (experiment) and 1 kHz (simulation). I: control; II: impending status; III: established status; IV: subtle status.

**Fig 4:**
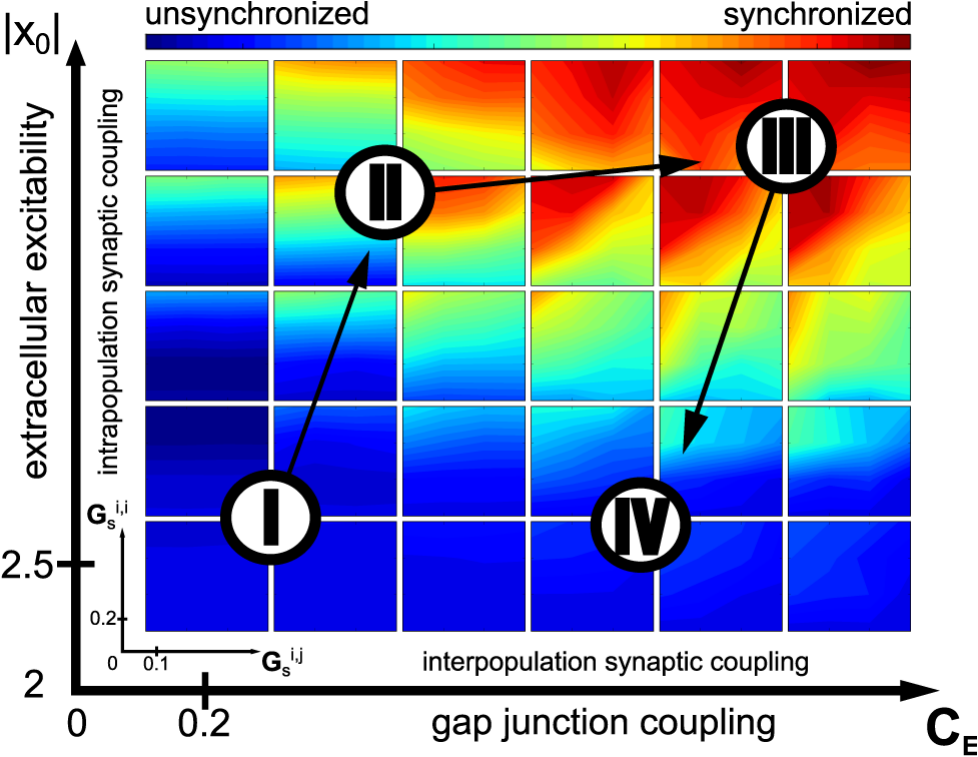
Parameter space during status epilepticus (SE). Color code represents the degree of synchronization within neural populations. Roman numbers indicate the phases of the SE corresponding to the observed dynamics following Fig 2. Arrows depict the trajectory of the sequence of theses phases during the whole SE event. Inner x- and y-axis are inter (G_s_^i,j^) and intra (G_s_^i,i^) population synaptic coupling strength. Outer x- and y-axes are gap junction coupling strength and excitability, respectively. Axis values are given for indicative purposes and do not portray biophysical units.

Status epilepticus (SE), i.e. uninterrupted seizure lasting at least 20 min, is a traumatic experience that can transform a non-epileptic brain into a brain displaying spontaneous seizures [32,33]. SE can be induced in rodents by injecting convulsant agents, such as kainic acid or pilocarpine [34,35]. It develops through 3 main phases called impending, established and subtle SE (**Fig 3**, timeseries II, III and IV, respectively), each of which being part of a continuum of electrophysiological fingerprints [36]. In the following, we retrace the route that neuronal networks follow during SE through a selected set of physiological parameters i.e. the extracellular excitability x_0_, inter- and intra-population synaptic coupling strengths G_s_^i,j^ and G_s_^i,i^ (glutamatergic and GABAergic for pyramidal cell and interneuron populations, respectively) and gap junction coupling strength C_E_ (**Fig 4**). We identify regimes according to the synchronization ratio between neurons within populations (color code), and map it to the characteristic phases observed experimentally (**Fig 3** and **Fig 4**, roman numbers). Since a single metric has not yet been identified to discriminate appropriately the different electrophysiological regimes observed during SE, the color code does not separate the SE states but rather offers a coarse map for orientation amongst the observed dynamical regimes. There are no clear demarcation lines between the dynamic regimes, since the transitions are more gradual than discrete.

#### Before status epilepticus

**Fig 5:**
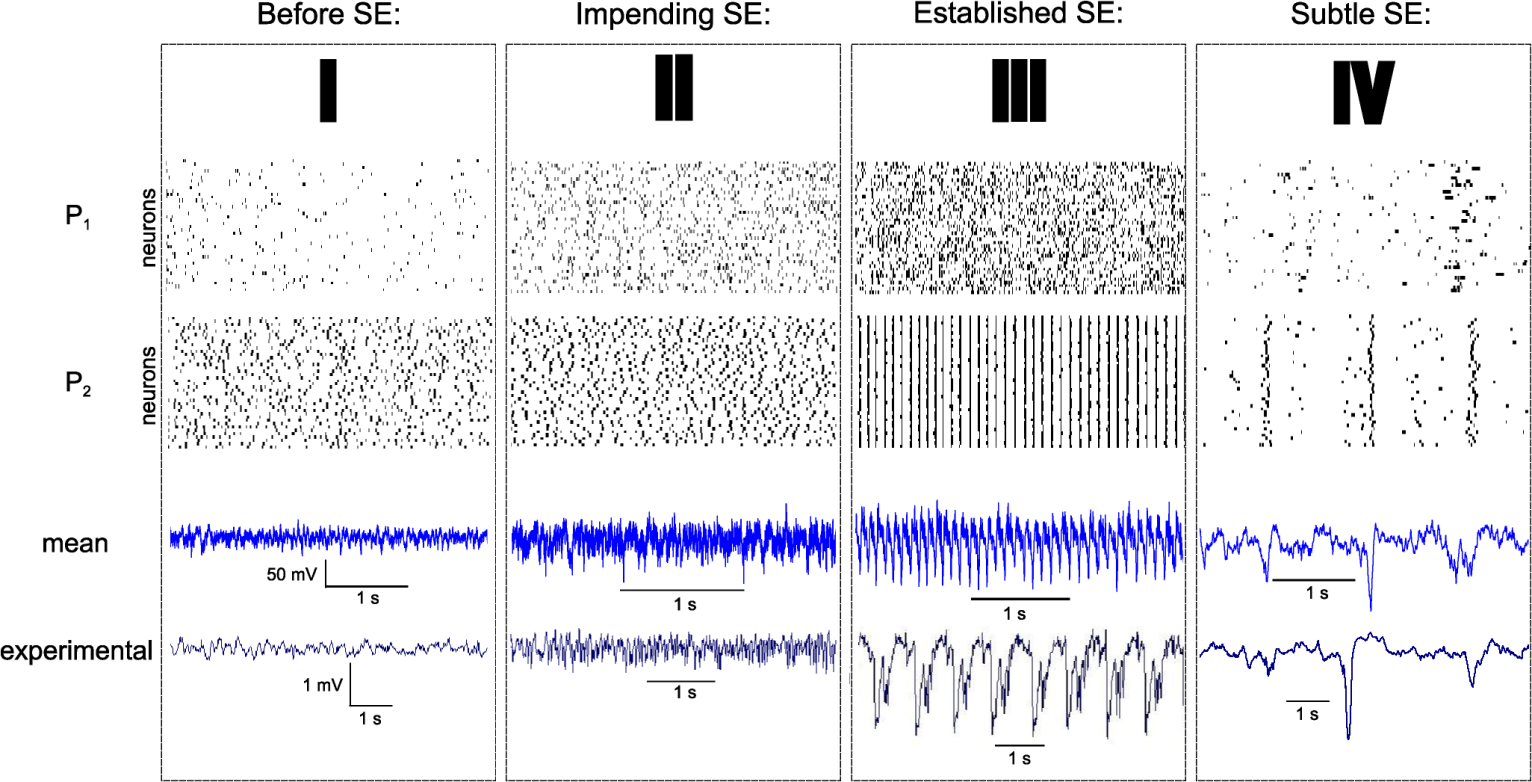
Population activities at different stages of status epilepticus in simulated and experimental traces. I, II, III, and IV correspond to the area of the parameter space spanned in Fig 3. P1 (excitatory) and P2 (inhibitory) are neural populations’ raster plots, with activation threshold at 0 mV. Black points are action potentials, of which the firing rate and synchronization properties change according to the different stages of SE. The mean activity is calculated as the sum of the average of P1 neurons and P2 neurons activity, with 80% and 20% contribution respectively. All experimental traces are recorded from the same rat and shown here before and after chemically-induced SE.

Experimentally, before the occurrence of SE, neuronal networks operate in a “normal” physiological regime, displaying typical broad-spectrum background activity (Fig 3, time series I) with a slight peak in the theta band (4–12 Hz) meaning that the animal is engaged in active motor behavior such as walking of exploratory sniffing. In the model, both excitatory and inhibitory populations display spontaneous unsynchronized neural activity during this regime (Fig 5, regime I). In particular, the firing rate of inhibitory neurons is larger than that of excitatory neurons, in keeping with the restraining action of interneurons; and excitatory neurons (from population 1) are quasi silent.

#### Impending status epilepticus

Experimentally, impending SE (or pseudo SE) is characterized by large amplitude excursions in the field potential, starting about half an hour after kainic acid injection (Fig 3, time series II and Fig 5, regime II). In the model, such activity can be generated by an increase of the absolute amplitude of the slow environmental variable *z*, raising excitability in both excitatory and inhibitory neuronal populations (as may happen experimentally when the extracellular concentration of potassium rises [11]) and awaking excitatory neurons (Fig 4 and Fig 5). Neurons from both populations still operate asynchronously but firing rates have increased and interneurons’ dampening role is diminished.

#### Established status epilepticus

Established (also called convulsive) stage of the SE *in vivo* is characterized by large rhythmic spikes in the 3–12 Hz frequency band, accompanied with faster discharges in the gamma range (30–80 Hz). Our simulations reveal that this epoch corresponds to rhythmic synchronized spiking of inhibitory neurons, while excitatory neurons display hyperactive activity at significantly higher frequencies and also increased synchronicity, albeit to a lesser degree than their inhibitory counterparts (Fig 5, regime III).

The frequency in the simulated trace is about 3 times higher than in the experimental time series recorded from a rat brain. Such frequency differences between model and experimental data can be attributed to the variability in the low frequency peak of the experimental data. Indeed, when kindling is used to trigger SE in rats, discharges in the 13 Hz range occurs, as in the model (S1 Fig). Our parameter space analysis suggests that only strong electrotonic coupling between neurons permits to enter such paroxysmal dynamics (Fig 4), supporting the idea that gap junctions between neurons are important for the hyper-synchronization process [37].

#### Subtle status epilepticus

The subtle phase is the last stage of the SE. It is characterized experimentally by reduced or absent fast discharges and a progressive shift from large spike waves towards interictal spike-like discharges. This phase gradually appears after the late convulsive phase, typically 10 to 20 hours after SE onset. Behaviorally, animals are not convulsing anymore and recover normal functions (exploration, feeding…). With regard to the populations’ activities (Fig 5, regime IV), our simulations support the notion that the 3–12 Hz sharp oscillations during the seizure (i.e. population rhythmic spiking observed in Fig 5, regime III) are generated by the same dynamic feature as the interictal spikes (Fig 5, regime IV); this feature being mostly carry out by a neuronal population composed of inhibitory cells shaping the spike-wave part, whereas the ictal fast oscillations are generated by the excitatory cells. We differentiate interictal spikes from spike-wave discharges when they appear at a frequency less than 1 Hz.

### C. Spontaneous seizure: From interictal to ictal activity

**Fig 6:**
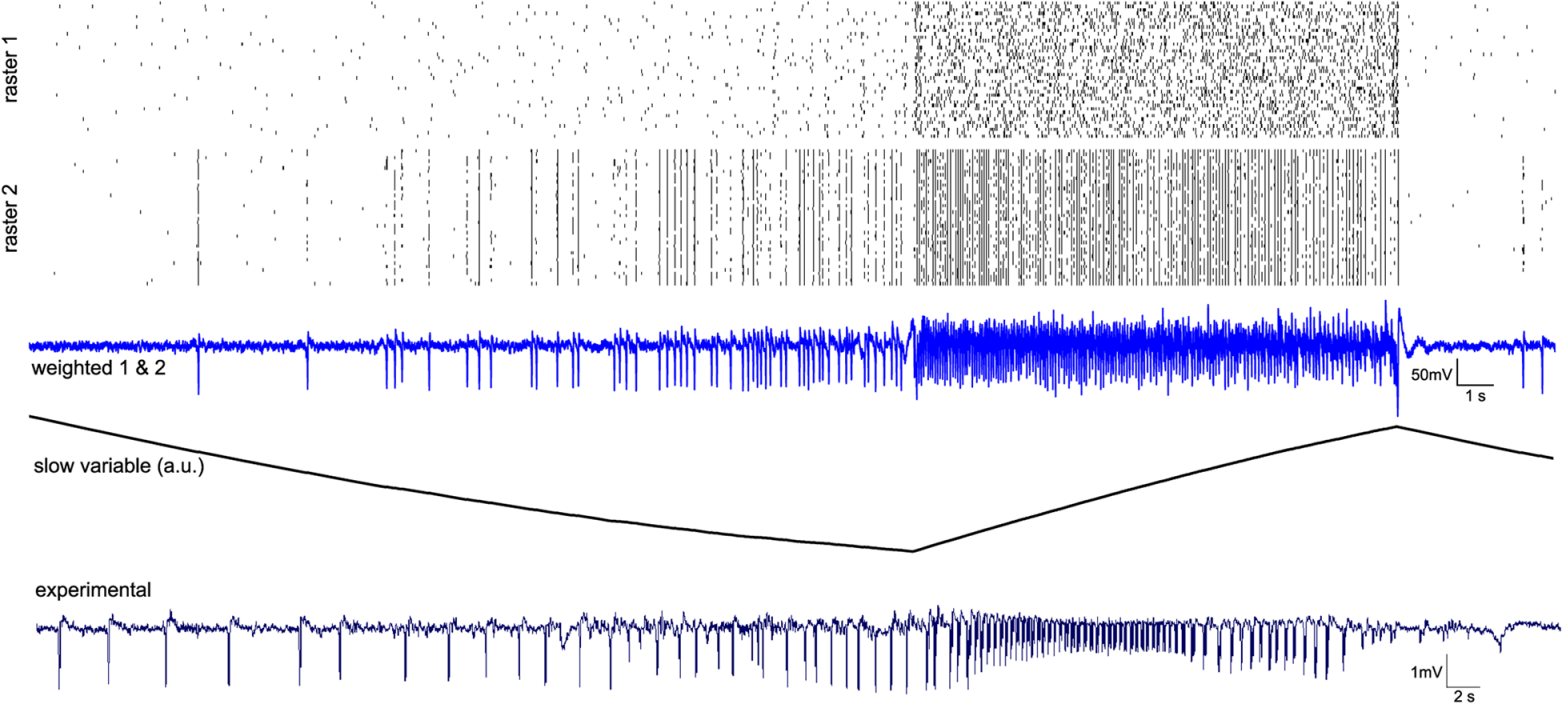
Activity of populations of excitatory and inhibitory ensembles, slow variable and mean time-series during a spontaneous seizure. Raster plots 1 & 2 display spikes of population 1 and 2 neurons, with activation threshold at 0 mV; third trace is the mean of the two populations with contribution of 80% from excitatory and 20% from inhibitory neurons; slow permittivity variable is the z variable (in arbitrary unit) from Eq 3, which trigger onset and offset of seizure; bottom plot is experimental data from rat scalp recording in vivo. Parameters: C_E_ = 1.0; x_0_ = 3.0; W^max^ = 0.4; G_s_^i,j^ = 0.2; G_s_^i,i^ = 0.1; r = 0.000004.

After SE, animals experience a latent period during which complex network reorganizations take place. During such period, although neuronal networks exhibit interictal-like activity [38], there are no spontaneous seizures. The latter occur during the chronic phase, a few days or weeks after SE. They are difficult to predict; the brain appears to operate “normally” before an abrupt change happens, characterized by 2 to 10-fold larger amplitude oscillations, which is the seizure. Our model reproduces the most important features of such transitions i.e. an abrupt fast firing discharge pattern at seizure onset, and a decrease of spike-wave frequency towards the end of seizure. We predict interictal spikes and spike-wave discharges are generated from synchronized activity of inhibitory neurons, and are affected by synaptic coupling strengths within and between the two populations of neurons. **Fig 6** displays a simulation of about a minute of activity in which a seizure takes place, together with its experimental counterpart. The model produces the different states of seizure evolution without any change of parameters; the states include pre-ictal population spikes, abrupt transitions to tonic firing, and seizure offset. Hysteresis effects have been predicted in the Epileptor [11] and are preserved in the coupled neuronal population dynamics relayed by the slow permittivity variable. As permittivity traces out its trajectory, seizure onset and offset occur at different values of permittivity and the two different neuronal spiking patterns of the populations may co-exist for the same permittivity value. These behaviors are characteristic for hysteresis.

**Fig 7:**
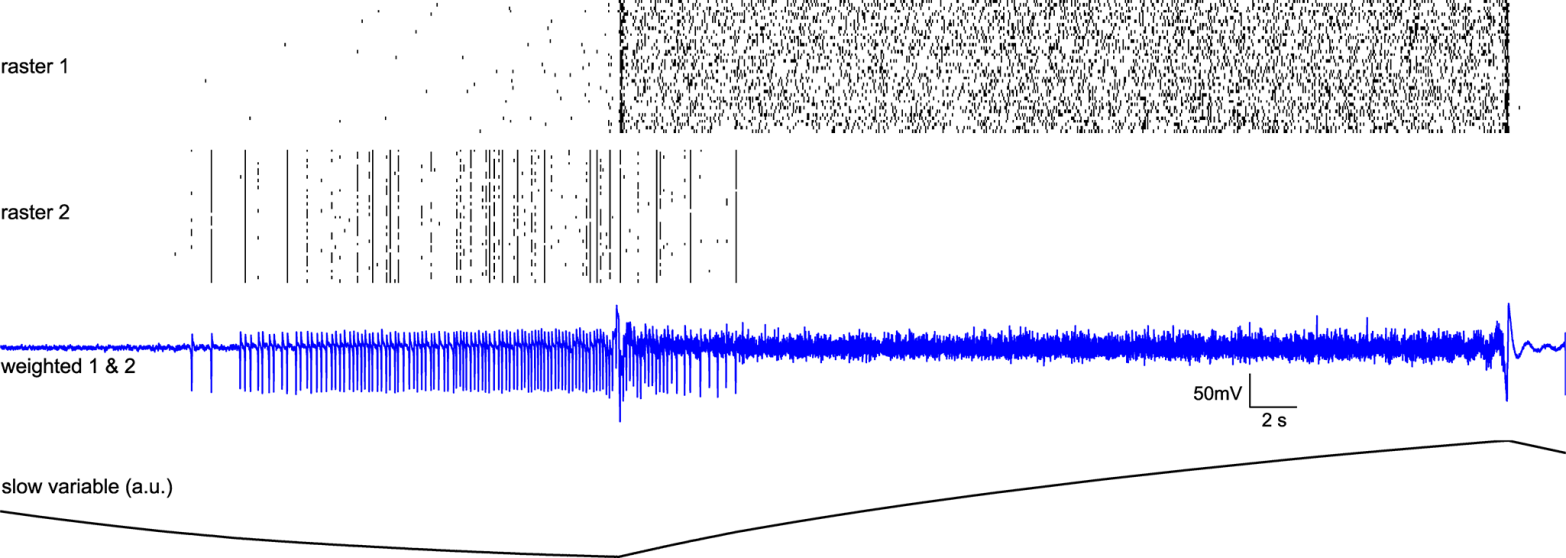
Simulated seizure-like event in the absence of glutamatergic and GABAergic synaptic couplings. Raster plots 1 & 2 display spikes of population 1 and 2 neurons, with activation threshold at 0 mV; third trace is the mean of the two populations with 80% contribution of excitatory ensembles and 20% contribution of inhibitory ensembles; slow permittivity variable is the z variable from Eq 3 (in arbitrary unit), mainly affected by excitatory cells, exerting a inhibitory effect on inhibitory cells upon exogenous factors; bottom plot is experimental data. Parameters: C_E_ = 0.8; x_0_ = 3.0;W^max^ = 0.2; G_s_^i,j^ = 0; G_s_^i,i^ = 0; r = 0.000004.

Synaptic coupling is also supposed to play a central role in seizure genesis. However, extracellular Ca^2+^ concentration nearly drops to zero during seizures, including in primates [39]. In the absence of extracellular Ca^2+^ seizures can occur in neuronal networks [40], following the general rules of seizure dynamics [11]. When glutamatergic and GABAergic synaptic couplings are removed in the model (G_S_^i,j^ = 0 in Eq 3.1), we still observe seizure-like events but the temporal features of the signal are slightly different (**Fig 7**), although hysteresis is maintained. In such conditions, inhibitory populations’ spikes and pyramidal fast discharges influence each other’s excitation through the extracellular environment, thus on slower timescale than spiking discharges. We propose a mechanism around seizure onset: the frequency of inhibitory population spikes increases until the excitatory population starts to fire (onset), and from there, inhibitory neurons reduce their activity to finally stop firing for the remainder of the event. Considering the temporal evolution of the slow variable in the deprived synaptic situation, we note that the inhibitory population’s activity is present when the variable is low. As soon as the excitatory lead takes place, the slow variable rises and quickly the inhibitory population becomes quiescent. This prediction would imply that the action of the excitatory activity on the extracellular medium acts as brake on synchronized inhibitory population’s spikes.

**Fig 8:**
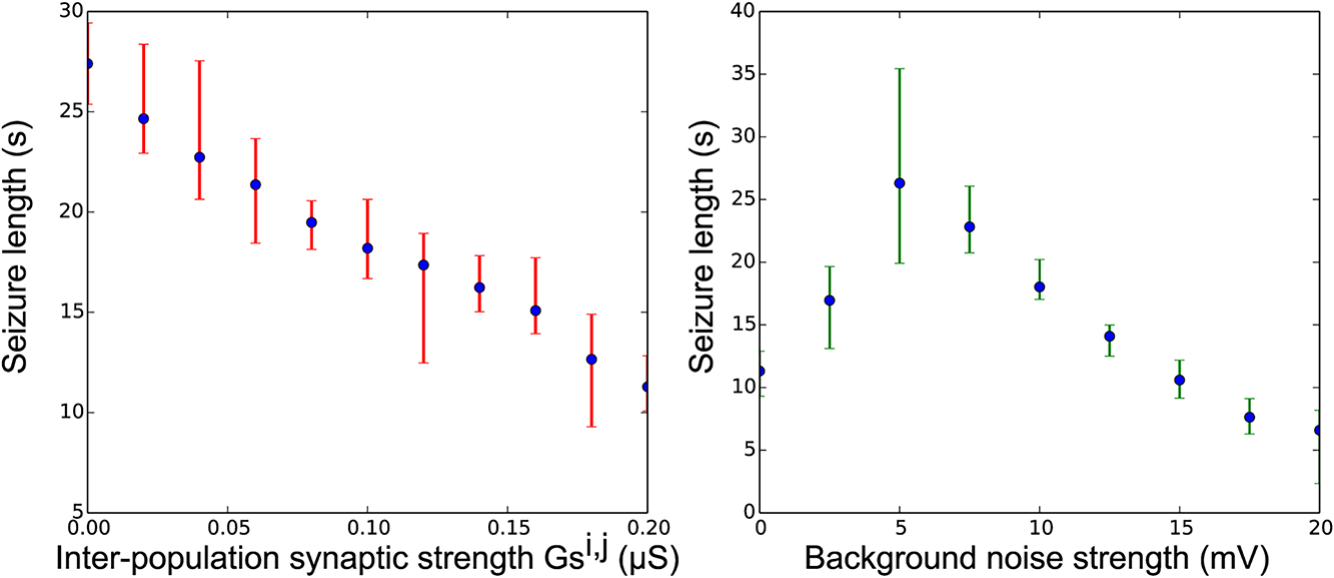
Seizure lengths according to inter-population coupling (left) and background noise (right). Simulations were performed to reproduce 15 minutes of activity for each parameter setting, resulting in 10 to 30 seizures per simulations. Mean, minimum and maximum seizure lengths were calculated from these generated time series by isolating the fast firing neurons’ activity and counting the time spent in up-state (threshold at -40 mV). Up-states of less than 5s were not considered as seizure and down-states of less than 3s between consecutive seizures were considered as being part of the same seizure. Noise is inserted in both excitatory and inhibitory neurons. Parameters: C_E_ = 0.8, x_0_ = 3.0; W_max_ = 0.2 (left plot); G_s_^i,j^ = 0.1 (right plot); G_s_^i,i^ = 0.1; r = 0.000004.

Running systematic simulations over coupling strength parameters, we also observe that seizure duration decreases as we increase inter-population coupling (**Fig 8**, left). This can be understood as follows: as inter-population coupling increases, the synaptic gain of GABA synapses coming from synchronized spiking of inhibitory cells increases, which reduces the activity in the excitatory cells, eventually leading to the destruction of the self-sustained epileptiform discharges. It is notable that the modification of inter-population coupling results in multi-scale effects on fast and slow time scales and, in particular, significantly affects slower time-scales such as seizure duration.

We finally performed simulations with different noise levels, as noise has a strong effect on neuronal network dynamics [41,42]. Our results support the idea that a noisy environment impacts epileptiform activity. In the context of epilepsy, the degree of stochasticity versus determinism in neural systems has been analyzed, but evidence for one or the other mechanism is still lacking [43]. With our model, low noise intensity in the spontaneous seizure configuration leads to long lasting, sharp onset and offset seizures with long periods between seizure-like activities. Increasing noise intensity results in more frequent but shorter seizure-like discharges (**Fig 8**, right), with less synchronized ensembles of neurons.

## Discussion

The role of extracellular mechanisms in epilepsy has become more prominent over the last decade [44,45]. Previous computational modeling studies incorporated selected features of extracellular environment (oxygenation, [K^+^]_o_…) in their mathematical formulation and investigated mechanisms of transitions between brain states [12–14,20]. Here we have adopted the Epileptor modeling approach of Jirsa and colleagues [11], in which multiple intra/extracellular factors are absorbed in a permittivity variable that influences population neural excitability on a time scale of several seconds. Permittivity may include various factors such as [K^+^]_o_ but also pH [46], calcium concentration [47,48], ATP resources [49], state of blood vessels [45], and oxygen availability [14,50]. Thus, instead of attempting to incorporate them all in a biophysical description resulting in an unmanageable complexity of simulation parameter tuning, they are lumped into the slow permittivity variable affecting neural excitability.

The link between the phenomenological Epileptor and a network model of coupled spiking neurons allows us to gain a deeper understanding of the robustness of the dynamic structures (in particular its phase flow topology) present in the Epileptor, when being subjected to biophysical constraints such as network connectivity or spike averaging. Gold standard Hodgkin Huxley equations [51] have many parameters and degrees of freedom, hence concerns exist with regard to parameter choices, in particular in light of the non-bijective nature of neuron dynamics (many parameter combinations can give rise to the same dynamics, see for instance [17]). Here we chose the reduced neuron models of Hindmarsh-Rose and Morris-Lecar as an intermediate step, which still allow us to pose questions on population averaging and coupling effects, but within a reduced parameter set and well-defined dynamics. These reduced neuron models [52,53] isolate conceptually the essential mathematical properties of excitation and inhibition from the electrochemical properties of sodium and potassium flows and thus provide a simpler mathematical description. In particular, the mathematical properties of the neuron models resemble those present in the Epileptor, which offers a starting point for the modeling process.

As full synchronization is approached with strong electrical coupling within a neuronal population, we demonstrated in **Fig 6** and **Fig 7** that seizure onset and offset are triggered in a synergistic manner between fast dynamics, typically neuron spikes, and a slower dynamical environment embedding multiple physiological factors. Uncoupled isolated neurons show, by construction, the same dynamic isomorphism, but then noise will drive the synchronization apart, which is not the case in the electrically coupled population. There, the dynamic range of behaviors survives the noise effects, in particular due to gap junction couplings (see **Fig 4** and **Fig 8**). Electrical coupling via gap junction is modeled here by the difference coupling $C_{E}\left({\bar{x}}_{i}-x_{i}\right)$ as known from electrical circuit theory. This mathematical representation could also be considered a linear approximation of field effects, which are a special case of ephaptic communication [54], but a rigorous mathematical representation thereof still needs to be developed.

Previous computational studies have highlighted the role of gap junctions for synchronization and epilepsy, in particular for gap junctions located on the axons of glutamatergic neurons [55]. Experimentally, there is evidence for gap junctions only between the dendrites of GABAergic interneurons, but despite numerous electron microscopic studies, there is as yet no ultrastructural evidence of gap junctions between somatodendritic domains of hippocampal pyramidal neurons [27]. Our study suggests that such electrical coupling is in particular responsible for the synchronization of inhibitory neurons, which then in turn give rise to the spike-wave part of interictal spikes. In contrast, most of the current literature, both from computational and experimental studies, supports the hypothesis that interictal discharges arise from synchronization of bursts of pyramidal neurons and interneurons [56–58]. Our results (as hypothesized in [11]), support the mechanism by which the large amplitude discharge of the spike-wave component arises from synchronized discharges of inhibitory interneurons only, and the slower wave component with its coincident high-frequency discharges is generated by excitatory neurons. This high frequency firing is consistent with more detailed computational studies [6,55], however the generation of the spike-wave component is different. Supporting our prediction, GABAergic neurons recorded during spike-wave events stopped firing during the fast discharge, and resumed firing when spike-wave events reoccurred during seizures [11]. Current clamp recordings revealed that they stopped firing during the fast discharge because they entered into depolarization block. There is obviously further experimental work to be performed to disambiguate these effects although some evidence is being accumulated [59]. The theoretical framework presented here should be able to aid in sharpening the investigations. Our scheme does not necessarily oppose previous studies, as the way interictal spikes are generated may vary along the course of epileptogenesis [38].

Several physiological mechanisms have been proposed and investigated to explain pathological activity in the context of epilepsy. Paroxysmal depolarization shifts (PDS) became more prominent as a potential mechanism to explain excitatory neural activity during interictal spikes and seizures [60]. PDS are strong neural depolarization blocks that lead to sustained bursting and maintained membrane depolarization with a very long-lasting return to baseline resting membrane potential (see Figure 4 in [61]). Our simulations support the view of such hyper-excited activity in excitatory ensembles during seizure, in which the permittivity in our model regulates excitability. However, the design of our model regarding inhibitory ensembles does not allow testing whether interneurons follow the same track of depolarization. Such behavior can be observed experimentally when GABAergic transmission becomes depolarizing [62–64], a phenomenon known to happen in early development [65].Future studies using more physiological inhibitory neuron models than our two-dimensional regular spiking Morris-Lecar models could possibly investigate this question more adequately. Another mechanism is cell swelling, which in astrocytes and neurons can affect cellular and network function [66], potentially increasing neural excitability and synchronizing populations of neurons [16,67]. Our results support the hypothesis that electrotonic coupling between neurons via gap junctions is increased in epileptic conditions [37,55]. Extracellular space shrinkage, conveying more surface contact between cells and thus favoring electric communication, has also been demonstrated experimentally in previous studies [68] and is compatible with our results concerning the involvement of permittivity and gap junction coupling in seizure genesis and evolution. Cell swelling together with extracellular alkalization or intracellular acidification [56], induced by trans-membrane movement of ions and water associated with bursting activity during paroxysmal depolarization shifts, also increases field interactions through exogenous communication [54]. Permittivity comprises such field effects as part of the slow variable *z*, but their role and relevance for neural activity is so far unknown. Our simulations in deprived synaptic conditions argue towards the idea that strong pyramidal cell activity would reduce interneuron activation by such coupling via the extracellular space. This “extracellular environmental disinhibition” may be negligible as compared to synaptic strengths in standard conditions, nevertheless leading to spike-waves with embedded fast discharges. It is important to note that the extracellular concentration of calcium drops to the 100 μM range *in vivo* during seizures in primates [39]. At this concentration, synaptic transmission is largely compromised [69], yet spike waves and fast discharges still occur, supporting the above-mentioned mechanism. Surprisingly, using our model with high noise level, the variance of this slow extracellular fluctuation variable is reduced. This result brings new possible insights about the mechanisms of seizure occurrence frequency shifts on long time scale in some types of epilepsy. For example, time between consecutive seizures often increase in patients enduring frequent seizures in childhood [70]. Following our results, this condition may be due to very slow decrease of background noise level (in the order of months and years) during development [71,72], as empirical evidence states that signal variability decreases with age [73].

### Conclusion

Approaches using dynamical system theory may provide guidance when deriving biophysical models of brain function and dysfunction. Here we used the abstract Epileptor model to guide the design of population models of coupled excitatory and inhibitory spiking neurons, which now allows studying the organization of spiking patterns as a function of coupling and excitability. We demonstrated that gap junction coupling plays the dominant role in synchronizing both neuron types, whereas the slow permittivity changes act rather as a slowly changing control parameter aiding in organizing the seizure progression. Our simulations support that the large amplitude discharge of spike-wave components of interictal and ictal population spikes arises from synchronized discharges of inhibitory interneurons only, which is not in line with current thinking in the literature, though finds support by recent empirical studies. Our approach does not rule out other physiological organizations giving rise to the same dynamics as described in the Epileptor; in fact, there is a large range of candidates. Thus although our findings may have validity only within a small range of physiological realizations, they nevertheless can give insight about experimental paradigms via simulations and analyses bridging the gap between neuronal spiking, network and abstract seizure evolution across large temporal scales.

## Supporting Information

S1 Fig 13 Hz discharges in a seizure recording of a rat with induced epilepsy via kindling. In this trace (right), the paroxysmal oscillation has a frequency higher than in the experimental and simulated signal displayed in this article, showing inter-individual diversity not taken in consideration in our model. Spectrum is shown on the left.
(EPS)

S1 Movie Animated motions of oscillations in the phase space and around the unit circle. (top)The reduced two-dimensional phase space of bursting (right) and regular spiking (left) populations during spontaneous activity at seizure onset. Nullclines are drawn in blue and green lines, of which the intersection is a fixed point. For each population, the left-most intersection is a stable fixed point and the right-most intersection is an unstable fixed point labeled by a filled red dot. Each neuron is represented as a filled blue dot, the mean of all neurons from the same phase space is filled in black. Positions are updated in time. (middle) For both phase spaces, the phase of each neuron is projected on its respective unit circle, from which the center is taken to be the red-filled dot from the phase plane above. Neurons, thus represented as oscillators, have different colors to facilitate differentiation. The black segment originating from the center of the unit circle directs towards the mean phase of the oscillators. The norm of this vector is what is referred in the manuscript as the Kuramoto order parameter (KOP). (bottom)The bottom graph displays the time serie of the whole system (i.e. the weighted sum of neurons membrane potentials from both populations) for the duration of the animated movie. A dot is walking on the time serie to indicate the current position of the display.
(AVI)
